# Structures of the first representatives of Pfam family PF06938 (DUF1285) reveal a new fold with repeated structural motifs and possible involvement in signal transduction

**DOI:** 10.1107/S1744309109050416

**Published:** 2010-03-05

**Authors:** Gye Won Han, Constantina Bakolitsa, Mitchell D. Miller, Abhinav Kumar, Dennis Carlton, Rafael J. Najmanovich, Polat Abdubek, Tamara Astakhova, Herbert L. Axelrod, Connie Chen, Hsiu-Ju Chiu, Thomas Clayton, Debanu Das, Marc C. Deller, Lian Duan, Dustin Ernst, Julie Feuerhelm, Joanna C. Grant, Anna Grzechnik, Lukasz Jaroszewski, Kevin K. Jin, Hope A. Johnson, Heath E. Klock, Mark W. Knuth, Piotr Kozbial, S. Sri Krishna, David Marciano, Daniel McMullan, Andrew T. Morse, Edward Nigoghossian, Linda Okach, Ron Reyes, Christopher L. Rife, Natasha Sefcovic, Henry J. Tien, Christine B. Trame, Henry van den Bedem, Dana Weekes, Qingping Xu, Keith O. Hodgson, John Wooley, Marc-André Elsliger, Ashley M. Deacon, Adam Godzik, Scott A. Lesley, Ian A. Wilson

**Affiliations:** a Joint Center for Structural Genomics, http://www.jcsg.org, USA; b Department of Molecular Biology, The Scripps Research Institute, La Jolla, CA, USA; c Program on Bioinformatics and Systems Biology, Burnham Institute for Medical Research, La Jolla, CA, USA; d Stanford Synchrotron Radiation Lightsource, SLAC National Accelerator Laboratory, Menlo Park, CA, USA; e Département de Biochimie, Faculté de Médecine, Université de Sherbrooke, Sherbrooke (Quebec), Canada; f Protein Sciences Department, Genomics Institute of the Novartis Research Foundation, San Diego, CA, USA; g Center for Research in Biological Systems, University of California, San Diego, La Jolla, CA, USA; h Photon Science, SLAC National Accelerator Laboratory, Menlo Park, CA, USA

**Keywords:** structural genomics, domain of unknown function, domain duplication, signaling, oxidative stress

## Abstract

The crystal structures of SPO0140 and Sbal_2486 were determined using the semiautomated high-throughput pipeline of the Joint Center for Structural Genomics (JCSG) as part of the NIGMS Protein Structure Initiative (PSI). The structures revealed a conserved core with domain duplication and a superficial similarity of the C-terminal domain to pleckstrin homology-like folds. The conservation of the domain interface indicates a potential binding site that is likely to involve a nucleotide-based ligand, with genome-context and gene-fusion analyses additionally supporting a role for this family in signal transduction, possibly during oxidative stress.

## Abstract 2

The crystal structures of SPO0140 and Sbal_2486 revealed a two-domain structure that adopts a novel fold. Analysis of the interdomain cleft suggests a nucleotide-based ligand with a genome context indicating signaling as a possible role for this family.

## 1. Introduction

To extend the structural coverage of proteins with uncharacterized biological function, we targeted Pfam protein family Pfam06938 (DUF1285), for which we determined the structures of two representative members. The *SPO0140* gene of *Silicibacter pomeroyi* DSS-­3, a marine α-proteobacterium, encodes a protein with a molecular weight of 21.2 kDa (residues 1–193) and a calculated iso­electric point of 5.7. The *Sbal_2486* gene of *Shewanella baltica* OS155, a psychrotropic marine γ-proteobacterium, encodes a protein with a molecular weight of 17.7 kDa (residues 1–157) and a calculated iso­electric point of 4.8.

## 2. Materials and methods

### 2.1. Protein production and crystallization

The clones for SPO0140 and Sbal_2486 were generated using the Polymerase Incomplete Primer Extension (PIPE) cloning method (Klock *et al.*, 2008 ▶). The gene encoding SPO0140 (GenBank YP_165412, gi:56695065, UniProt Q5LWU5) was amplified by polymerase chain reaction (PCR) from *Silicibacter pomeroyi* DSS-3 genomic DNA using *PfuTurbo* DNA polymerase (Stratagene) and I-­PIPE (Insert) primers (forward primer, 5′-ctgtacttccagggcATGAGCGG­ACAAAAGCCTGTGAAACC-3′; reverse primer, 5′-aattaagtcgcgt­taGCCGCGCTCCAGCTCCTCGACCGTCATC-3′; target sequence in upper case) that included sequences for the predicted 5′ and 3′ ends. The expression vector pSpeedET, which encodes an amino-terminal tobacco etch virus (TEV) protease-cleavable expression and purification tag (MGSDKIHHHHHHENLYFQ/G), was PCR-amplified with V-PIPE (Vector) primers (forward primer, 5′-taacgc­gacttaattaactcgtttaaacggtctccagc-3′; reverse primer, 5′-gccctggaagtac­aggttttcgtgatgatgatgatgatg-3′). The V-PIPE and I-PIPE PCR products were mixed to anneal the amplified DNA fragments together. *Escherichia coli* GeneHogs (Invitrogen) competent cells were transformed with the V-PIPE/I-PIPE mixture and dispensed onto selective LB–agar plates. The cloning junctions were confirmed by DNA sequencing. Expression was performed in a selenomethionine-containing medium at 310 K with suppression of normal methionine synthesis. At the end of fermentation, lysozyme was added to the culture to a final concentration of 250 µg ml^−1^ and the cells were harvested and frozen. After one freeze–thaw cycle, the cells were homogenized in lysis buffer [50 m*M* HEPES pH 8.0, 50 m*M* NaCl, 10 m*M* imidazole, 1 m*M* tris(2-carboxyethyl)phosphine–HCl (TCEP)] and the lysate was clarified by centrifugation at 32 500*g* for 30 min. The soluble fraction was passed over nickel-chelating resin (GE Healthcare) pre-equilibrated with lysis buffer, the resin was washed with wash buffer [50 m*M* HEPES pH 8.0, 300 m*M* NaCl, 40 m*M* imidazole, 10%(*v*/*v*) glycerol, 1 m*M* TCEP] and the protein was eluted with elution buffer [20 m*M* HEPES pH 8.0, 300 m*M* imidazole, 10%(*v*/*v*) glycerol, 1 m*M* TCEP]. The eluate was buffer-exchanged with TEV buffer (20 m*M* HEPES pH 8.0, 200 m*M* NaCl, 40 m*M* imidazole, 1 m*M* TCEP) using a PD-10 column (GE Healthcare) and incubated with 1 mg TEV protease per 15 mg of eluted protein. The protease-treated eluate was run over nickel-chelating resin (GE Healthcare) pre-equilibrated with HEPES crystallization buffer (20 m*M* HEPES pH 8.0, 200 m*M* NaCl, 40 m*M* imidazole, 1 m*M* TCEP) and the resin was washed with the same buffer. The flowthrough and wash fractions were combined and concentrated to 13.9 mg ml^−1^ by centrifugal ultrafiltration (Millipore) for crystallization trials. SPO0140 was crystallized by mixing 200 nl protein solution with 200 nl crystallization solution and using a 50 µl reservoir using the nanodroplet vapor-diffusion method (Santarsiero *et al.*, 2002 ▶) with standard Joint Center for Structural Genomics (JCSG; http://www.jcsg.org) crystallization protocols (Lesley *et al.*, 2002 ▶). The crystallization reagent consisted of 20%(*v*/*v*) ethanol and 100 m*M* Tris pH 8.5. Glycerol was added to a final concentration of 17%(*v*/*v*) as a cryoprotectant. A rod-shaped crystal of approximate dimensions 0.08 × 0.02 × 0.02 mm was harvested after 29 d at 277 K for data collection. Initial screening for diffraction was carried out using the Stanford Automated Mounting (SAM) system (Cohen *et al.*, 2002 ▶) at the Stanford Synchrotron Radiation Lightsource (SSRL, Menlo Park, California, USA). The diffraction data were indexed in the tetragonal space group *P*4_3_2_1_2.

The gene encoding Sbal_2486 (GenBank YP_001050848.1, gi:126174699, UniProt A3D5G6) was amplified from *Shewanella baltica* OS155 genomic DNA. Using the PIPE method (Klock *et al.*, 2008 ▶), the initial clone was generated by using I-PIPE (Insert) primers (forward primer, 5′-ctgtacttccagggcATGGAAAAGATGA­CTGACAGTATTCAAC-3′; reverse primer, 5′-aattaagtcgcgttaCT­GCTCATTTAGATCAGATAAATTG-3′; target sequence in upper case) that included sequences for the predicted 5′ and 3′ ends. Cloning, expression and purification were performed as described above for SPO0140. Crystals obtained from the full-length construct were not suitable for data collection. Bioinformatic predictions suggested that a 12-residue N-terminal truncation might produce better diffracting crystals than the full-length (157 residues) wild-type protein. Cloning attempts were initiated to generate nested truncations in steps of four residues around this prediction for a truncated sequence. Additionally, C-terminal truncations (NB these were not part of the prediction to improve crystallization) were attempted. Truncation clones were successfully generated for the construct boundaries 9–157, 17–157, 21–157, 1–153, 1–149, 1–145 and 1–141. These constructs were screened in parallel for solubility, crystallization and diffraction (Table 1 ▶). No clone was obtained for the initially proposed truncation (13–157) construct. The other three N-terminal truncations produced soluble protein that led to harvestable crystals that were of higher quality and diffracted better than the crystals of the full-length protein. The only C-terminal truncation that produced soluble protein was that for residues 1–153. This construct also led to harvestable crystals, which again were of higher quality and diffracted better than the full length construct. The improvement was less than that observed for the N-terminal truncation constructs. By making several truncation constructs and empirically testing all of them, we found a construct that was better suited to crystallization and structure determination. A crystal of the 9–157 construct was used for structure solution. The primers used to generate the 9–157 truncation clone by PIPE mutagenesis were I-­PIPE (Insert) forward primer 5′-ctgtacttccagggcCAACACACACTCAAACAATTCGCCG­CCG-3′ and reverse primer 5′-gccctggaa­gtacaggttttcgtgatgatgat­gatgatg-3′ (Klock *et al.*, 2008 ▶). Purified Sbal_2486 was concentrated to 20 mg ml^−1^ by centrifugal ultrafiltration (Millipore) for crystallization trials. Sbal_2486 was crystallized by mixing 200 nl protein solution and 200 nl crystallization solution and using a 50 µl reservoir volume using the nanodroplet vapor-diffusion method with standard JCSG crystallization protocols. The crystallization reagent consisted of 20%(*v*/*v*) 2-propanol, 20%(*w*/*v*) PEG 4000 and 0.1 *M* sodium citrate pH 5.6. Ethylene glycol (1,2-ethanediol) was added to a final concentration of 10%(*v*/*v*) as a cryo­protectant. A rhombohedral crystal of approximate size 0.1 × 0.1 × 0.1 mm was harvested after 20 d at 277 K for data collection. Initial screening for diffraction was carried out using the SAM system and an X-ray microsource (Miller & Deacon, 2007 ▶) installed at SSRL. The diffraction data were indexed in the orthorhombic space group *P*2_1_2_1_2_1_.

### 2.2. Data collection, structure solution and refinement

For SPO0140, multiple-wavelength anomalous diffraction (MAD) data were collected on beamline BL11-1 at SSRL at wavelengths corresponding to the inflection (λ_3_-2re3), high-energy remote (λ_1_-­2re3) and peak (λ_2_-2re3) of a selenium MAD experiment. The data sets were collected at 100 K with a MAR Mosaic 325 mm CCD detector (Rayonix) using the *Blu-Ice* (McPhillips *et al.*, 2002 ▶) data-collection environment. The MAD data were integrated and reduced using *MOSFLM* (Leslie, 1992 ▶) and were scaled using the program *SCALA* (Collaborative Computational Project, Number 4, 1994 ▶). Selenium-substructure solution and phasing were performed with *SHELXD* (Sheldrick, 2008 ▶) and *autoSHARP* (Bricogne *et al.*, 2003 ▶) with a mean figure of merit of 0.49 for 12 selenium sites (NB there are five unique Se sites per chain, but SeMet131 adopts two different con­formations resulting in two partial occupancy sites). Automatic model building was performed with *ARP*/*wARP* (Cohen *et al.*, 2004 ▶). Model completion and refinement were performed with *Coot* (Emsley & Cowtan, 2004 ▶) and *REFMAC*5.2 (Winn *et al.*, 2003 ▶) using the remote (λ_1_-2re3) data set. The refinement included experimental phase restraints in the form of Hendrickson–Lattman coefficients from *SHARP*, NCS restraints (positional weight 0.5 and thermal weight 2.0) and TLS refinement with one TLS group per chain. Data-reduction and refinement statistics for SPO0140 are summarized in Table 2 ▶.

For Sbal_2486, MAD data were collected on beamline 8.2.2 at the ALS at wavelengths corresponding to the inflection (λ_2_-2ra9), low-energy remote (λ_3_-­2ra9) and peak (λ_1_-2ra9) of a selenium MAD experiment. The inflection and remote data were collected first using an interleaved protocol with a 10° wedge size over a total sweep of 100° and the peak data were then collected in a 130° sweep. The data sets were collected at 100 K with a Quantum 315 CCD detector (ADSC). The MAD data were integrated and reduced using *MOSFLM* and were scaled with the program *SCALA*. Selenium-substructure solution and phasing were performed with *SHELXD* and *autoSHARP* with a mean figure of merit of 0.48 for a single selenium site. Automatic model building was performed with *ARP*/*wARP*. Model completion and refinement were performed with *Coot* (Emsley & Cowtan, 2004 ▶) and *REFMAC*5.2 (Murshudov *et al.*, 1999 ▶) using the peak (λ_1_-2ra9) data set. The refinement included experimental phase restraints in the form of Hendrickson–Lattman coefficients from *SHARP* and restrained anisotropic ADP refinement. Data-reduction and refinement statistics for Sbal_2486 are summarized in Table 3 ▶.

### 2.3. Validation and deposition

**Figure 1:**
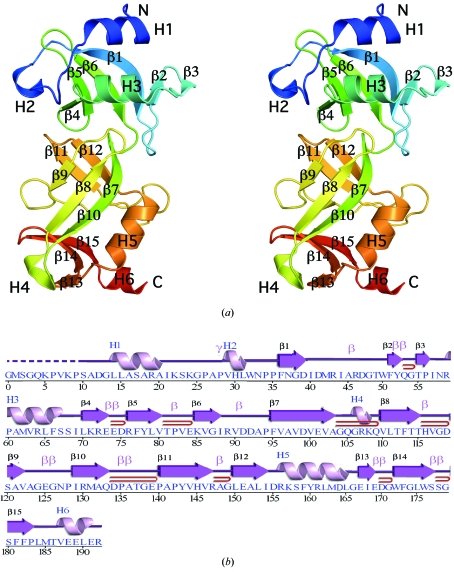
Crystal structure of SPO0140 from *Silicibacter pomeroyi*. (*a*) Stereo ribbon diagram of the SPO0140 monomer (chain *A*) color-coded from the N-terminus (blue) to the C-­terminus (red). Helices (H1–H6) and β-strands (β1–β15) are indicated. (*b*) Diagram showing the secondary-structure elements of SPO0140 superimposed on its sequence in accordance with *PDBsum* (http://www.ebi.ac.uk/pdbsum). For SPO0140, the α-helices (H1, H3, H5 and H6), 3_10_-helices (H2 and H4), β-strands (β1–β15), β-turns (β) and γ-turns (γ) are indicated. The β-hairpins are indicated by red loops. Residues not included in the final model are indicated with a dashed line.

The quality of the crystal structures was analyzed using the JCSG Quality Control server (http://smb.slac.stanford.edu/jcsg/QC). This server processes the coordinates and data through a variety of validation tools including *AutoDepInputTool* (Yang *et al.*, 2004 ▶), *MolProbity* (Davis *et al.*, 2007 ▶), *WHAT IF* v.5.0 (Vriend, 1990 ▶), *RESOLVE* (Terwilliger, 2003 ▶) and *MOLEMAN2* (Kleywegt, 2000 ▶), as well as several in-house scripts, and summarizes the output. Protein quaternary-structure analysis used the *PISA* server (Krissinel & Henrick, 2007 ▶). Fig. 1 ▶(*b*) was adapted from an analysis using *PDBsum* (Laskowski *et al.*, 2005 ▶) and all others were prepared with *PyMOL* (DeLano Scientific). Atomic coordinates and experimental structure factors for SPO0140 at 2.5 Å resolution and Sbal_2486 at 1.4 Å resolution have been deposited in the PDB and are accessible under codes 2re3 and 2ra9, respectively.

## 3. Results and discussion

### 3.1. Overall structure

The crystal structure of SPO0140 (Fig. 1 ▶) was determined to 2.5 Å resolution using the MAD method. Data collection, model and refinement statistics are summarized in Table 2 ▶. The final model included two protomers (residues 10–192 for chain *A*, residues 10–193 for chain *B*), one glycerol molecule and 186 water molecules in the asymmetric unit. No electron density was observed for residues Gly0 (which remained at the N-terminus after cleavage of the purification tag), SeMet1–Pro9 in chains *A* and *B* and Gly193 in chain *A*. Side-chain atoms of Lys108, Thr137 and Glu139 in chain *A* and Ser10, Lys108, Gln133, Thr137 and Glu139 in chain *B* had poorly defined electron density and were omitted from the model. The Matthews coefficient (*V*_M_; Matthews, 1968 ▶) was 3.0 Å^3^ Da^−1^ and the estimated solvent content was 59.0%. The Ramachandran plot produced by *MolProbity* (Davis *et al.*, 2007 ▶) showed that 97.0% of the residues were in favored regions, with no outliers.

The crystal structure of Sbal_2486 was determined to 1.4 Å resolution using the MAD method. Data collection, model and refinement statistics are summarized in Table 3 ▶. The final model included a monomer of 127 residues, seven ethylene glycol molecules, one sodium ion and 231 water molecules in the asymmetric unit. No electron density was observed for residues Gly0 (which remained at the N-­terminus after cleavage of the purification tag), Gln9–Cys28 and Glu156–Gln157. The Matthews coefficient (*V*_M_; Matthews, 1968 ▶) was 2.6 Å^3^ Da^−1^ and the estimated solvent content was 52.5%. The Ramachandran plot produced by *MolProbity* showed that 97.6% of the residues were in favored regions, with no outliers.

**Figure 2:**
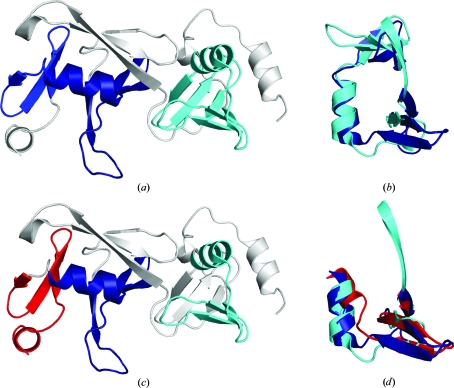
Structural representation of the repeated β_3_αβ_3_ and β_3_α motifs in SPO0140. (*a*, *b*) SPO0140 contains two β_3_αβ_3_ motifs. (*a*) Ribbon diagram of SPO0140 (PDB code 2re3; residues 10–192; gray) showing the relative orientation of the two β_3_αβ_3_ motifs (residues 34–92, cyan; residues 126–186, blue) in the structure and (*b*) the same repeats superimposed. (*c*, *d*) SPO0140 contains three β_3_α motifs. (*c*) Ribbon diagram of SPO0140 as in (*a*) showing the relative orientation of the three β_3_α motifs (residues 36–67, cyan; residues 129–164, blue; residues 167–192, red) and (*d*) the three β_3_α motifs superimposed.

SPO0140 is an α/β protein comprising two domains (Fig. 1 ▶). The N-­terminal domain (residues 10–93) consists of a β-meander–α-­helix–β-meander core (residues 24–92), with the two β-meanders hydrogen bonding along the first and sixth strands to form a twisted mixed six-stranded β-sheet. Two N-terminal helices (H1 and H2) pack against the sheet and complete this domain. The same β_3_αβ_3_ unit (β1–3, H3 and β4–6; residues 36–88) is encountered again in the C-terminal domain (β10–12, H5 and β13–15; residues 129–83), with an additional three-stranded meander (β7–9; residues 95–122) packing perpendicularly against the second meander to form a β-sandwich (Fig. 1 ▶ *a*). Both repeats share the same overall fold and topology and their structural comparison is considered to be significant by different alignment methods. Both *FATCAT* (Ye & Godzik, 2003 ▶) and *DALI* (Holm *et al.*, 2008 ▶) show a statistically significant similarity, with the *FATCAT* alignment yielding a C^α^ r.m.s.d. of 3.0 Å over 51 residues (Figs. 2 ▶ *a* and 2 ▶ *b*) and a sequence identity of 8% (*DALI Z* score 4.0, r.m.s.d. of 3.6 Å over 48 residues, 15% sequence identity). The main difference involves the orientation of the β3 meanders with respect to each other, resulting in different hydrogen-bonding patterns (*i.e.* the two meanders are connected in the N-terminal domain but form two separate sheets in the C-terminal domain).

Three smaller β_3_–α-helix (β_3_α) repeats (residues 36–67, 129–164 and 167–192) were also identified in the structure and can be generated from the first repeat by an approximate 90° anti­clockwise rotation along the helix axis between the first and second motif and a clockwise 90° rotation between the second and third (Fig. 2 ▶ *c*). Although the C-terminal helix is both shorter and differently oriented with respect to the meander when compared with the first two β_3_α repeats (Fig. 2 ▶ *d*), this unit may constitute a minimal supersecondary structure motif for this protein.

**Figure 3:**
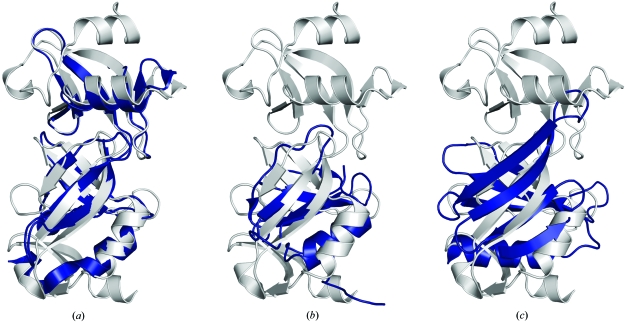
Structural comparisons between DUF1285 homologs and PH-like domains. Ribbon diagram showing the superposition of SPO0140 (PDB code 2re3, residues 10–192; gray) with, in blue, (*a*) Sbal_2486 (PDB code 2ra9, residues 29–155), another DUF1285 homolog, (*b*) a prokaryotic PH-like domain PA2021 (PDB code 1ywy, residues 23–96) and (*c*) a canonical prokaryotic PH domain (PDB code 3hsa, residues 22–179).

The two domains of Sbal_2486 (residues 29–84 and 85–155) show close structural similarity to SPO0140 (C^α^ r.m.s.d. of 2.8 Å over 127 residues with a sequence identity of 25%). Structural elements missing from Sbal_2486 involve SPO0140 helices H1 and H2 and part of strand β1, as well as the C-terminal β-meander (strands β13–β15) and helix H6 (Fig. 3 ▶ *a*). Thus, although the β_3_αβ_3_ repeat described for SPO0140 is present in the N-terminal domain of Sbal_2486, the loss of the C-terminal meander results in a truncated version of this repeat in the second domain. The hypothesis of the fold having originated *via* duplication of the β_3_αβ_3_ repeat is supported by homolog sequence analysis, which shows the N-terminal domain to be more strongly conserved than the C-terminal domain. Helix H1 and the β7–β9 meander can then be viewed as additions to the core repeat that help to stabilize each domain. Alternatively, the fold can also be viewed as consisting of a repetition of β_3_ units followed either by an α-helix or a turn. The β_3_-turn repeats (strands β4–β6 and β7–β9) are conserved in both structures, with the conservation of the β7–β9 meander arguing in favor of this possibility. In the case of the β_3_α motifs, three repeats are encountered in longer homologs, such as SPO0140, and two are found in shorter versions, such as Sbal_2486.

Searches with *FATCAT* (Ye & Godzik, 2003 ▶), *DALI* (Holm *et al.*, 2008 ▶) or *SSM* (Krissinel & Henrick, 2004 ▶) revealed no significant hits for the N-terminal domain of SPO0140 or Sbal_2486. In all three methods, the closest structural neighbor of the SPO0140 C-terminal domain is the PA2021 protein (PDB code 1ywy; Y. C. Lin, G. Liu, Y. Shen, A. Yee, C. H. Arrowsmith & T. Szyperski, unpublished work) from *Pseudomonas aeruginosa*, with a C^α^ r.m.s.d. of 3.4 Å over 67 residues and a sequence identity of 12% (Fig. 3 ▶ *b*). The similarity extends over strands β7–β9 and the second β_3_α motif (strands β10–β12 and helix H5). *SCOP* classifies PA2021 as a pleckstrin-homology (PH) domain-like barrel. However, both this similarity and the similarity of the same region of SPO0140 to the canonical prokaryotic PH domain (PDB code 3hsa; Joint Center for Structural Genomics, unpublished work; Fig. 3 ▶ *c*) are not statistically significant in all of the algorithms employed (*DALI Z* score 0.4, r.m.s.d. of 4.6 Å over 42 residues, 10% sequence identity; *SSM Z* score 1.4, *P* score 0, r.m.s.d. of 3.2 Å over 53 residues, 4% sequence identity; Laskowski *et al.*, 2005 ▶) and the overall topologies of the two domains are different, with the PH domain containing an additional N-terminal strand and a longer helix in a different orientation with respect to the DUF1285 β3-helix meander (β10–β12). These considerations led us to classify the SPO0140 and Sbal_2486 structures as a new fold, an assessment that is supported by a preliminary *SCOP* analysis (Alexey Murzin, personal communication).

SPO0140 crystallized with two monomers (*A* and *B*) in the asymmetric unit. The largest packing interface buries ∼810 Å^2^ of solvent-accessible surface per monomer and involves mainly helix H1, strand β1 and the H3–β4 loop from the N-terminal domain and helix H4 and strands β14–β15 from the C-terminal domain. However, packing analysis using *PISA* (Krissinel & Henrick, 2007 ▶) suggests that this would not be a stable dimerization interface. The results of analytical size-exclusion chromatography (anSEC) support the assignment of a monomer as the quaternary state in solution for SPO0140.

Sbal_2486 crystallized with one monomer in the asymmetric unit and *PISA* again suggested a monomer as the most probable oligomeric form. The largest packing interface buries ∼760 Å^2^ of solvent-accessible surface area but is not predicted to be stable for complex formation. However, the results of anSEC coupled with static light scattering (SLS) for Sbal_2486 indicate a dimeric form in solution under the test conditions. This suggests that either the observed crystal-packing interface is stable or the oligomerization state is dependent on the buffer conditions, forming a dimer in the anSEC buffer but disassociating in the crystallization solution prior to crystal formation.

### 3.2. Analysis of a conserved cavity

Analysis of SPO0140 and Sbal_2486 using the *CastP* server (Binkowski *et al.*, 2003 ▶) revealed that the largest cavity occurs at the interdomain interface. Surface-conservation analysis using *ConSurf* (Landau *et al.*, 2005 ▶) showed this to be the largest and most highly conserved contiguous region in the DUF1285 family; it includes loops β1–β2, β6–β7 and strand β4 from the N-terminal domain and strands β10–β12 from the C-terminal domain. No strictly conserved residues are found in this region among DUF1285 homologs, leading us to propose that this region might act as a binding site, but not one that exhibits catalytic activity.

A search against a database of cognate binding sites using *IsoCleft* (Najmanovich *et al.*, 2008 ▶), a graph-matching algorithm that searches for similarities in both geometry and chemical composition, identified shared features between the inter-domain cleft of SPO0140 and sugar, phosphate and purine-binding proteins [PDB codes 1pwh (Yamada *et al.*, 2003 ▶), 1dqa (Istvan *et al.*, 2000 ▶), 1dm3 (Modis & Wierenga, 2000 ▶), 1gpe (Wohlfahrt *et al.*, 1999 ▶), 1v0j (Beis *et al.*, 2005 ▶), 1hwy (Smith *et al.*, 2001 ▶), 2vfs (Forneris *et al.*, 2008 ▶) and 1q6p (Scapin *et al.*, 2003 ▶)]. Similar hits (adenosylcobalamin, heme, dideoxy sugars, NAD, thiamine diphosphate) were obtained for Sbal_2486 (Supplementary Table S1^1^). These similarities, combined with the high conservation observed in this region of the DUF1285 structures and genome-context analysis (see below), indicate that a nucleotide-based ligand may bind along the interdomain interface.

### 3.3. Gene-fusion and genome-context analysis

The DUF1285 homolog from *Marinobacter* sp. ELB17 includes a phosphoglycerate mutase domain (Pfam PF00300) as part of the gene preceding DUF1285. This domain is further annotated as belonging to the subgroup of SixA phosphohistidine phosphatases (IPR004449). In prokaryotes, transcriptional profiling has shown that expression of phosphoglycerate mutase is increased under conditions of oxidative stress (Nodop *et al.*, 2008 ▶), while the SixA phosphohistidine phosphatase in *E. coli* has been implicated in signal transduction under conditions of anaerobic respiratory growth (Matsubara & Mizuno, 2000 ▶). However, as this is the only example of such a domain fusion encountered in the DUF1285 family, the scope for functional speculation is limited.

Several genes predicted (http://string.embl.de) to have functional associations with SPO0140 are located in the same genome neighborhood. Nudix hydrolases are observed in the genome neighborhood of the majority of DUF1285 homologs, including SPO0140. Nudix hydrolases are pyrophosphatases that control the cellular concentrations of a variety of nucleoside diphosphate derivatives, including nucleoside diphosphates and triphosphates and their oxidized forms, dinucleoside polyphosphates, NADH and other signaling compounds (Kraszewska, 2008 ▶). In plants, these enzymes have been implicated in oxidative signaling (Jambunathan & Mahalingam, 2006 ▶; Mahalingam *et al.*, 2006 ▶), including the maintenance of cellular redox homeostasis (Ge *et al.*, 2007 ▶) and resistance to exogenous reactive oxygen species (Tong *et al.*, 2009 ▶). In bacteria, nucleotide-based second messengers are involved in a range of signaling functions (Pesavento & Hengge, 2009 ▶), including the oxidative stress response (Johnstone & Farr, 1991 ▶). Similarly, the DUF1285 family might carry out a signaling function related to oxidative stress, possibly through binding to a small nucleotide derivative.

The SPO0140 protein family (DUF1285, PF06938) is encountered mainly in proteobacteria and contains around 200 sequence homologs which vary between 150 and 250 residues in length. Availability of more DUF1285 sequences and structures might shed light on the evolutionary history of this intriguing protein family. The information presented here, in combination with further biochemical and bio­physical studies, should yield valuable insights into the functional role of SPO0140 and Sbal_2486. Models for SPO0140 and Sbal_2486 homologs can be accessed at http://www1.jcsg.org/cgi-bin/models/get_mor.pl?key=2re3A and http://www1.jcsg.org/cgi-bin/models/get_mor.pl?key=2ra9A, respectively.

Additional information about SPO0140 and Sbal_2486 is available from TOPSAN (Krishna *et al.*, 2010 ▶) at http://www.topsan.org/explore?PDBid=2re3 and http://www.topsan.org/explore?PDBid=2ra9, respectively.

## 4. Conclusions

The first structural representatives of the DUF1285 family revealed a novel fold consisting of repeated motifs. Sequence-conservation and genome-context analysis suggests a signaling role possibly involving binding to a small nucleotide derivative under conditions of oxidative stress.

## Supplementary Material

PDB reference: SPO0140 from *Silicibacter pomeroyi* DSS-3, 2re3

PDB reference: Sbal_2486 from *Shewanella baltica*, 2ra9

Supplementary material file. DOI: 10.1107/S1744309109050416/wd5122sup1.pdf

## Figures and Tables

**Table 1:** Summary of expression and diffraction screening results for Sbal_2486 constructs Diffraction screening was carried out by collecting two diffraction images 90° apart and evaluating the resulting images for resolution, diffraction strength, ice rings and spot quality.

Construct	Soluble	No. of crystals screened	Best screening resolution† (Å)	Median screening resolution (Å)	Best crystal-quality score‡	Best spot-quality score§	Average spot-quality score
1–157	+	213	3.0	6.5	6	8	6.7
1–153	+	19	2.8	5.8	7	8	8.0
1–149	−						
1–145	−						
1–141	−						
9–157	+	68	2.2	4.2	10	9	8.1
17–157	+	31	2.2	3.9	10	10	8.4
21–157	+	34	2.5	4.5	8	9	7.9

† Note that crystals of the full-length construct (1–157) were screened using the synchrotron beam at SSRL, while crystals of the truncated constructs were screened using the X-ray microsource. The resolution from synchrotron data collection is typically 0.6–1.2 Å better than the screening resolution obtained using the microfocus sealed-tube system. The resolution from synchrotron-screened crystals is more comparable to the final resolution after data collection.

‡ Crystal quality is an overall integer score of 0–10 that is assigned to assess the suitability of the crystal for data collection, with 10 being the best-quality crystals and 0 corresponding to no diffraction. The score is based on resolution, spot quality, diffraction strength, single *versus* multiple lattices, ice-ring pathology and other factors. Only crystals with scores of 5 or better are saved for further evaluation and data collection.

§ Spot quality is assigned as an integer score of 0–10, with 10 corresponding to nice clean spots, 5–6 corresponding to mostly elongated or anisotropic spots, 1–3 corresponding to split spots and 0 corresponding to extreme streaks or powder-like patterns.

**Table 2:** Summary of crystal parameters, data collection and refinement statistics for SPO0140 (PDB code 2re3) Values in parentheses are for the highest resolution shell.

	λ_1_-2re3	λ_2_-2re3	λ_3_-2re3
Space group	*P*4_3_2_1_2		
Unit-cell parameters	*a* = *b* = 75.37, *c* = 182.69		
Data collection			
Wavelength (Å)	0.9184	0.9791	0.9794
Resolution range (Å)	29.5–2.50 (2.56–2.50)	29.5–2.50 (2.56–2.50)	29.5–2.50 (2.56–2.50)
No. of observations	141130	141301	141452
No. of unique reflections	19038	19111	19093
Completeness (%)	99.9 (100.0)	99.9 (100.0)	99.9 (100.0)
Mean *I*/σ(*I*)	12.3 (2.9)	12.0 (2.6)	12.3 (2.9)
*R*_merge_ on *I*† (%)	12.9 (76.0)	13.4 (80.9)	12.5 (71.8)
Model and refinement statistics			
Resolution range (Å)	29.5–2.50		
No. of reflections (total)	18959‡		
No. of reflections (test)	972		
Completeness (%)	99.7		
Data set used in refinement	λ_1_-2re3		
Cutoff criterion	|*F*| > 0		
*R*_cryst_§	0.215		
*R*_free_¶	0.258		
Stereochemical parameters			
Restraints (r.m.s.d. observed)			
Bond lengths (Å)	0.014		
Bond angles (°)	1.56		
Average isotropic *B* value (Å^2^)	25.4		
ESU based on *R*_free_ value†† (Å)	0.28		
Protein residues/atoms	367/2949		
Water/other solvent molecules	186/1		

† *R*_merge_ = 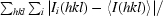 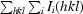.

‡ The number of unique reflections that were used in refinement is typically slightly less than the total number that were integrated and scaled. Reflections are excluded owing to systematic absences, negative intensities and rounding errors in the resolution limits and unit-cell parameters.

§ *R*_cryst_ = 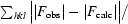 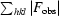, where *F*_calc_ and *F*_obs_ are the calculated and observed structure-factor amplitudes, respectively.

¶ *R*_free_ is the same as *R*_cryst_ but for 5.1% of the total reflections chosen at random and omitted from refinement.

†† Estimated overall coordinate error (Collaborative Computational Project, Number 4, 1994 ▶; Cruickshank, 1999 ▶).

**Table 3:** Summary of crystal parameters, data collection and refinement statistics for Sbal_2486 (PDB code 2ra9) Values in parentheses are for the highest resolution shell.

	λ_1_-2ra9	λ_2_-2ra9	λ_3_-2ra9
Space group	*P*2_1_2_1_2_1_		
Unit-cell parameters (Å)	*a* = 38.41, *b* = 62.29, *c* = 73.25		
Data collection			
Wavelength (Å)	0.9795	0.9798	1.0000
Resolution range (Å)	29.9–1.40 (1.44–1.40)	29.9–1.40 (1.44–1.40)	29.8–1.40 (1.44–1.40)
No. of observations	172763	130260	115916
No. of unique reflections	35222	34664	32638
Completeness (%)	99.6 (98.5)	98.2 (91.5)	92.6 (67.8)
Mean *I*/σ(*I*)	16.3 (2.7)	14.1 (2.1)	16.7 (1.9)
*R*_merge_ on *I*† (%)	5.8 (61.3)	6.0 (62.9)	4.6 (58.4)
Model and refinement statistics			
Resolution range (Å)	29.9–1.40		
No. of reflections (total)	35168[Table-fn tfn10]		
No. of reflections (test)	1764		
Completeness (%)	99.4		
Data set used in refinement	λ_1_-2ra9		
Cutoff criterion	|*F*| > 0		
*R*_cryst_[Table-fn tfn11]	0.162		
*R*_free_[Table-fn tfn12]	0.196		
Stereochemical parameters			
Restraints (r.m.s.d. observed)			
Bond lengths (Å)	0.014		
Bond angles (°)	1.52		
Average isotropic *B* value (Å^2^)	20.1		
ESU based on *R*_free_ value[Table-fn tfn13] (Å)	0.06		
Protein residues/atoms	127/1032		
Water/other solvent molecules	231/8		

† *R*_merge_ = 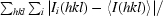 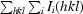.

‡ The number of unique reflections that were used in refinement is typically slightly less than the total number that were integrated and scaled. Reflections are excluded owing to systematic absences, negative intensities and rounding errors in the resolution limits and unit-cell parameters.

§ *R*_cryst_ = 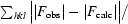 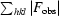, where *F*_calc_ and *F*_obs_ are the calculated and observed structure-factor amplitudes, respectively.

¶ *R*_free_ is the same as *R*_cryst_ but for 5.0% of the total reflections chosen at random and omitted from refinement.

†† Estimated overall coordinate error (Collaborative Computational Project, Number 4, 1994 ▶; Cruickshank, 1999 ▶).

## Footnotes

1 Supplementary material has been deposited in the IUCr electronic archive (Reference: WD5122).
