# Supplementary material for: Structures of the first representatives of Pfam family PF06938 (DUF1285) reveal a new fold with repeated structural motifs and possible involvement in signal transduction
Source: Acta Crystallogr Sect F Struct Biol Cryst Commun. 2010 Mar 5;66(Pt 10):1218–25. doi: 10.1107/S1744309109050416 (PMC2954208; doi:10.1107/S1744309109050416)
Supplement: Supplementary file 1 [file f-66-01218-sup1.pdf]

Table S1 *IsoCleft* (Najmanovich *et al.*, 2008) output for (A) SPO0140 (PDB id: 2RE3) and (B) Sbal_2486 (2RA9). Nc: number of atoms in common[1]; TSS: Tanimoto similarity score[2]; Z-score[3], P-value[4].

PDB id	Nc	TSS	Z-score	P-value
(A)				
1PWH	37	0.237	3.25	2.73E-02
1DQA	36	0.211	3.08	3.39E-02
1DM3	36	0.232	3.08	3.39E-02
1GPE	36	0.182	3.08	3.39E-02
1V0J	36	0.178	3.08	3.39E-02
1HWY	36	0.220	3.08	3.39E-02
2VFS	35	0.183	2.90	4.20E-02
1Q6P	35	0.213	2.90	4.20E-02
(B)				
1EEX	35	0.161	3.46	5.85E-01
1K7Y	34	0.160	3.27	5.98E-01
1U8X	33	0.198	3.08	3.39E-02
1GGE	33	0.192	3.08	3.39E-02
1TZF	33	0.262	3.08	3.39E-02
1ZPD	33	0.246	3.08	3.39E-02

[1] The number of atoms in common represents the largest subset of atoms from one cleft which are in equivalent relative geometric positions to atoms in the second cleft. Any given atom is additionally of the same atom type (hydrogen bond donor, acceptor, hydrophobic, etc) as its equivalent in the second cleft. In other words, the two clefts can be superimposed based on the detected chemical and geometric similarities.

[2] The Tanimoto Similarity Score is a normalized measure of similarity calculated as Nc/(N_A+N_B-Nc), where Na represents the number of atoms defining the query binding site (2RE2 or 2RA9) and Nb represent that of the target binding sites present in the IsoCleft Finder Database.

[3] The Z-score is calculated in the standard way as (Nc-<Nc>)/Std(Nc), where <Nc> is the average number of atoms in common for the comparison of the given query cleft against the whole IsoCleft Finder Database and Std(Nc) represents the standard deviation.

[4] P-values are calculated by fitting the distribution of Z-scores to an extreme value distribution as described in (Laskowski *et al.*, 2005).

References

Laskowski, R. A., Watson, J. D. & Thornton, J. M. (2005). *J. Mol. Biol.* **351**, 614-626.

Najmanovich, R., Kurbatova, N. & Thornton, J. (2008). *Bioinformatics* **24**, i105-111.
